# Early predictors of one-year mortality in patients over 65 presenting with ANCA-associated renal vasculitis: a retrospective, multicentre study

**DOI:** 10.1186/s12882-018-1102-3

**Published:** 2018-11-09

**Authors:** Dimitri Titeca-Beauport, Alexis Francois, Thierry Lobbedez, Dominique Guerrot, David Launay, Laurence Vrigneaud, Maité Daroux, Celine Lebas, Boris Bienvenu, Eric Hachulla, Momar Diouf, Gabriel Choukroun

**Affiliations:** 1 Department of Nephrology, Dialysis and Transplantation, Amiens University Hospital, F-80054 Amiens, France; 2 Department of Nephrology, Caen University Hospital, Caen, France; 3 Registre de Dialyse Péritonéale de Langue Française, Pontoise, France; 4 Department of Nephrology, Rouen University Hospital, Rouen, France; 5 INSERM, U1096 Rouen, France; 6 University of Lille, U995 Lille, France; 7 Lille Inflammation Research International Center (LIRIC), Lille, France; 8 Inserm, U995 Lille, France; 9 Département de Médecine Interne et Immunologie Clinique, CHU Lille, Lille, France; 10 Centre national de Référence Maladies Systémiques et Auto-immunes Rares (Sclérodermie Systémique), Lille, France; 11 Department of Nephrology and Internal Medicine, Valenciennes General Hospital, Valenciennes, France; 12 Department of Nephrology, Duchenne Hospital, Boulogne-sur-Mer, France; 13 Department of Nephrology, Calmette Hospital, Lille University Hospital, Lille, France; 14 Department of Internal Medicine, Caen, France; 15 Normandie Univ, UNICAEN, INSERM, COMETE, Caen, France; 16 Clinical Research and Innovation Directorate, Amiens University Hospital, Amiens, France

**Keywords:** ANCA, Elderly, Glomerulonephritis, Infection, Mortality

## Abstract

**Background:**

The risk of early death is particularly high in patients over the age of 65 presenting with antineutrophil cytoplasmic antibody (ANCA)-associated renal vasculitis. We hypothesized that by combining disease severity markers, a comorbidity index and serious adverse event reports, we would be able to identify early predictors of one-year mortality in this population.

**Methods:**

We performed a multicentre, retrospective study in the nephrology and internal medicine departments of six tertiary hospitals in northern France. A total of 149 patients (median [interquartile range (IQR)] age: 72.7 [68.5–76.8] years) presenting with ANCA-associated vasculitis and renal involvement were included between January 2002 and June 2015. The primary endpoint was the one-year mortality rate.

**Results:**

Renal function was severely impaired at presentation (median [IQR] peak serum creatinine (SCr): 337 [211–522] μmol/l), and 45 patients required dialysis. The Five-Factor Score (FFS, scored as + 1 point for each poor prognostic factor (age > 65 years, cardiac symptoms, gastrointestinal involvement, SCr ≥150 μmol/L, and the absence of ear, nose, and throat involvement)) was ≥3 in 120 cases. The one-year mortality rate was 19.5%. Most of the deaths occurred before month 6, and most of these were related to severe infections. In a univariate analysis, age, a high comorbidity index, a performance status of 3 or 4, a lack of co-trimoxazole prophylaxis, early severe infection, and disease activity parameters (such as the albumin level, haemoglobin level, peak SCr level, dialysis status, and high FFS) were significantly associated with one-year mortality. In a multivariable analysis, the best predictors were a high FFS (relative risk (RR) [95% confidence interval (CI)] = 2.57 [1.30–5.09]; *p* = 0.006) and the occurrence of a severe infection during the first month (RR [95%CI] = 2.74 [1.27–5.92]; *p* = 0.01).

**Conclusions:**

When considering various disease severity markers in over-65 patients with ANCA-associated renal vasculitis, we found that an early, severe infection (which occurred in about a quarter of the patients) is a strong predictor of one-year mortality. A reduction in immunosuppression, the early detection of infections, and co-trimoxazole prophylaxis might help to reduce mortality in this population.

**Electronic supplementary material:**

The online version of this article (10.1186/s12882-018-1102-3) contains supplementary material, which is available to authorized users.

## Background

Antineutrophil cytoplasmic antibody (ANCA)-associated vasculitis (AAV) is a systemic form of small vessel polyangiitis. Over 75% of patients with granulomatosis with polyangiitis (GPA), microscopic polyangiitis (MPA) or eosinophilic granulomatosis with polyangiitis are positive for circulating ANCA [1, 2].

AAV is primarily a disease of the elderly, with a mean age at diagnosis of 63 for patients with GPA and 66 for patients with MPA [3]. The incidence peaks at between 65 and 74 years of age, with 52.9 cases per million in the general population [4]. Renal involvement (characterized by focal, necrotizing, pauci-immune glomerulonephritis) is seen in over 70% of patients with AAV [5].

In patients over the age of 65, myeloperoxidase (MPO) ANCAs are preponderant. Renal function is generally severely impaired, and renal replacement therapy is frequently required. However, the clinical presentation is much the same as in younger patients [3]. Most deaths occur within the first year after diagnosis, and the two factors that best predict a poor prognosis in AAV are severe renal failure and age over 65 [6].

Patients over 65 with renal impairment at time of diagnosis are therefore particularly at risk of early death, since they have a higher risk of infection and reduced tolerance to immunosuppressive agents [7].

The objective of the present study was thus to identify early predictors for one-year mortality in a high-risk population of patients over 65 presenting with AAV and renal involvement.

## Patients and methods

### Study design

We performed a multicentre, retrospective study in the nephrology and internal medicine departments of four university hospitals and two tertiary hospitals in northern France. Patients aged 65 or more and presenting with AAV and inaugural renal involvement from January 2002 to June 2015 were included on the basis of registry data in the different centres.

The diagnosis of AAV with renal involvement was defined by acute renal impairment with proteinuria (> 300 mg/day) and/or haematuria (> 10/mm^3^) a positive ANCA assay (indirect immunofluorescence and/or an antigen-specific immunoassay), and (if available) a renal biopsy confirming the presence of pauci-immune glomerulonephritis. The time of diagnosis was defined as the date of the first ANCA-positive assay.

### Data collection

Patients were entered into the study from when a diagnosis of AAV with renal involvement was established. All data concerning the patients’ diagnosis and follow-up were extracted retrospectively from medical records.

At presentation, each patient’s age, gender, bodyweight and any history of hypertension, diabetes mellitus, chronic pulmonary disease, cardiovascular disease, malignancy and/or other comorbid conditions were recorded. The Charlson Comorbidity Index (CCI) was calculated from each patient’s data [8]. Baseline general condition (ECOG Performance Status, weight loss, fever, etc.), extrarenal manifestations, the Birmingham Vasculitis Activity Score (BVAS) were recorded. The 2009 Five-Factor Score (FFS) was calculated for each patient, with + 1 point for each poor prognostic factor (age > 65 years, cardiac symptoms, gastrointestinal involvement, stabilized peak serum creatinine (SCr) ≥150 μmol/L, and the absence of ear, nose, and throat (ENT) involvement) [9]. Clinical biochemistry data such as the serum C-reactive protein and albumin levels, the leukocyte count, the haemoglobin level, the baseline peak SCr level (in a pre-dialysis sample, in cases of dialysis), the urine protein-to-creatinine ratio, haematuria, and the type (proteinase 3 (PR3)/MPO) and level of ANCAs were recorded at diagnosis. Induction treatments of AAV (including steroids, cyclophosphamide (CYC), rituximab (RTX) and plasma exchange) were recorded. When performed, renal biopsies were rated according to Berden et al.’s prognostic classification [10].

During the study period, we recorded the glomerular filtration rate (calculated with the Modification of Diet in Renal Disease equation), dialysis, specific treatments and their dose levels, anti-infectious prophylaxis, the number and types of severe infection (defined as infections requiring hospitalization or intravenous antibiotics), leukopenia, cardiovascular events, and the above-mentioned clinical biochemistry parameters at month (M)1, M6, M12 and M24.

Remission was defined as the disappearance of clinically active disease and a stabilization of (or improvement in) renal function for at least 4 weeks. Relapse was defined as the reappearance of clinical symptoms and/or organ involvement prompting the intensification of immunosuppressive therapy. The time of first relapse was defined as the date when the immunosuppressive therapy was intensified. The last follow-up corresponded to the patient’s death or the last visit before the end of the study (June 30th, 2016). Hence, all included patients participated in the study for at least 12 months. The study protocol was approved by the local independent ethics committee (Amiens, France; reference: TB/LR/2016–88).

### Study endpoints

The primary endpoint was the one-year mortality rate. The secondary endpoints were the onset of end-stage renal disease (ESRD) and relapse.

### Statistical analysis

The patients’ characteristics were summarized as the frequency (%) for categorical variables and as the median and interquartile range [IQR] for continuous variables. Risk factors for one-year mortality were evaluated using univariate and multivariable log-binomial regression models, and the results were expressed as the relative risk (RR) [95% confidence interval (CI)] and the *p* value. Survival was assessed using the Kaplan–Meier method. The median follow-up time was calculated using the reverse Kaplan-Meier method. Risk factors for relapse and ESRD were investigated with univariate and multivariable competing-risks regression models by applying Fine and Gray’s method and calculating the hazard ratio (HR) [95%CI]. The cumulative incidence curves for ESRD and relapse were analysed in a competing risks regression model. The threshold for statistical significance was set to *p* < 0.05 in all univariate and multivariable analyses. All statistical analyses were performed using SAS software (version 9.4, SAS Institute Inc., Cary, NC) and R software (version 3.2.3, URL http://www.R-project.org/).

## Results

### The study population

Overall, 149 patients were included in the study and analyzed. The median [IQR] age at diagnosis was 72.7 [68.5–76.8], and 54 (36%) patients were over the age of 75. MPO-ANCAs were detected in 101 (68%) patients, and PR3-ANCAs were detected in 46 (31%) patients. Two patients had non-specific ANCAs. Eighty (54%) patients were diagnosed with MPA, 60 (40%) were diagnosed with GPA, and 9 (6%) were diagnosed with renal limited vasculitis (according to the Chapel Hill Consensus Conference nomenclature) [2]. The median [IQR] peak SCr level was 337 μmol/l [211–522], and 45 (30%) patients required dialysis at presentation. Renal biopsies were available for 131 patients. Pulmonary disease was the most common extrarenal manifestation and affected 67 (45%) patients, including 36 cases with diffuse alveolar haemorrhage. The median [IQR] BVAS was 18 [14–22], and 120 (81%) patients had an FFS ≥3. The baseline demographic and clinical data are summarized in Table 1.

**Table 1:** Baseline demographic and clinical data

Age (years)	72.7 [68.5–76.8]
Female	72 (48)
Comorbidities	
Hypertension	99 (66)
Diabetes mellitus	18 (12)
Cardiovascular diseases	21 (14)
Malignancy	16 (11)
Charlson Comorbidity Index	4 [3–5]
Performance Status	
0–2	129 (87)
3–4	20 (13)
AAV type	
MPO/PR3	101/46
GPA	60 (40)
MPA	80 (54)
RVL	9 (6)
Renal involvement	
Peak SCr (μmol/l)	337 [221–522]
uPCR (g/day)	1.8 [0.9–2.6]
Dialysis	45 (30)
Renal biopsy	131 (88)
Focal	31
Crescentic	38
Mixed	36
Sclerotic	26
Inflammatory parameters	
C-reactive protein (mg/l)	88 [19–151]
Serum albumin (g/l)	28 [22–32]
Leukocyte count (× 10^9^/l)	9.2 [7.1–12.9]
Haemoglobin (g/dl)	9.4 [8.3–10.5]
Organ involvement	
Cutaneous	12 (8)
Ocular	8 (5.3)
Ear, nose, throat	27 (18)
Pulmonary	67 (45)
Cardiac	8 (5.3)
Gastrointestinal	11 (7.3)
Musculoskeletal	41 (27.5)
Nervous system	27 (18)
BVAS	18 [14–22]
Five-Factor Score ≥ 3	120 (81)

Data are quoted as n (%) median and interquartile range. *AAV* anti-neutrophil cytoplasmic antibody, *ANCA* associated vasculitis, *MPO* myeloperoxidase, *PR3* proteinase 3, *MPA* microscopic polyangiitis, *GPA* granulomatosis with polyangiitis, *RVL* renal-limited vasculitis, *SCR* serum creatinine, *uPCR* urinary protein to creatinine ratio, *BVAS* Birmingham Vasculitis Activity Score

### Treatments

One hundred and forty-six patients received oral high-dose induction corticosteroids (1 mg/kg/day), and 117 of these received a median of 3 methylprednisolone pulses. One hundred and thirteen patients (76%) received CYC (intravenously in 96% of cases). The mean initial and one-month cumulative doses of CYC were respectively 11.0 ± 3.1 mg/kg/pulse and 25.6 ± 12.2 mg/kg. When considering the remaining patients, 11 (7.4%) received RTX, 20 (14.8%) received corticosteroids alone, one received azathioprine (AZA) and another received mycophenolate mofetil (MMF). Plasmapheresis was performed in 40 cases, including 18 individuals with pulmonary-renal syndrome. Prophylaxis with co-trimoxazole (CTZ) was administered in only 85 cases. Corticosteroids were progressively tapered after the first month, with a mean daily dose at M6 of 13.7 ± 9.1 mg. Of the 120 patients alive after 12 months, 109 were receiving maintenance treatment. Ninety-seven patients were still on oral steroids (mean daily dose: 8.8 ± 5.9 mg). The most commonly prescribed immunosuppressant was AZA (in 41 patients), followed by MMF (in 24) and RTX (in 19). Other drugs were administered much less frequently (e.g. methotrexate in 4 patients and CYC in 4 patients).

### Complications and patient survival

Eight patients were lost to follow-up after a median (range) time period of 76.9 months (24–127). The estimated median [95%CI] follow-up time was 69.3 months [53.4–76.0]. In all, 52 patients died, with 29 (56%) deaths related to infection, 7 (13%) related to cardiovascular disease, 4 related to malignancies and 4 directly related to active vasculitis. The estimated one, three and five-year survival rates [95%CI] were respectively 80% [74–87], 76% [70–84] and 74% [67–82].

Sixteen (11%) patients displayed leukopenia (< 4000 cells/mm^3^), and thirty-nine (26%) patients experienced a severe infection at some point during the first month of care. In total, 42% of the study population experienced at least one serious infectious complication during the first six months of care. The lungs, urinary tract, and abdomen were the most common infection sites, and Gram-negative bacteria and staphylococci were predominant.

**Fig. 1:**
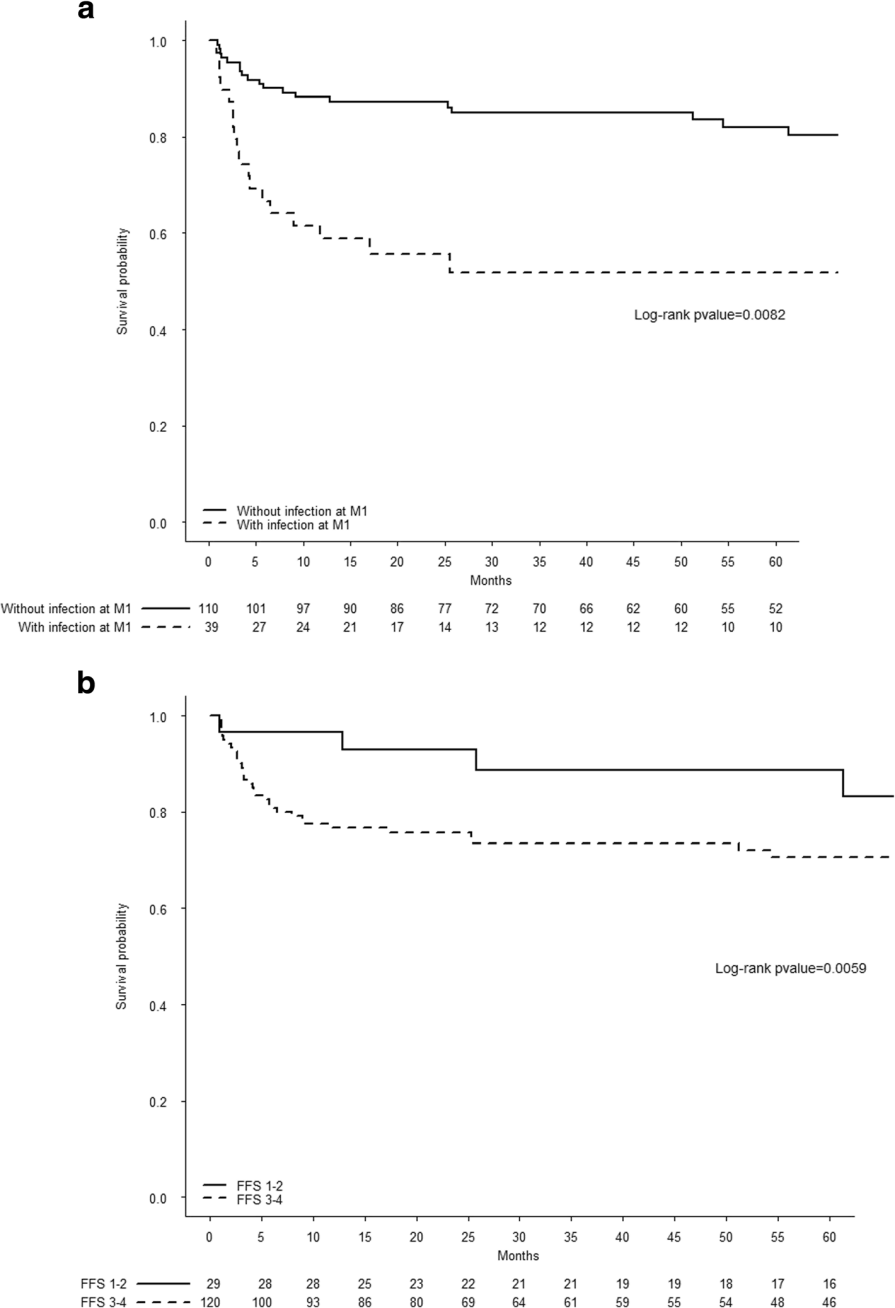
Kaplan-Meier curves for five-year survival, as a function of the independent predictors of a poor one-year outcome: **a** infections during the first month of care, and **b** the Five-Factor Score

Overall, 29 patients died during the first year of care, giving a one-year mortality rate of 19.5%. Twenty-four of these deaths occurred during the first six months, and 19 (70.3%) were related to infectious disease (mostly septic shock; for details, see Additional file 1: Table S1). In a univariate analysis, age, the CCI, a performance status of 3 or 4, a lack of CTZ prophylaxis, and disease activity parameters (such as albuminaemia, haemoglobin level, peak SCr level, and dialysis status) were significantly associated with one-year mortality (Table 2). In the multivariable model, a high FFS (RR [95%CI] = 2.57 [1.30–5.09]; *p* = 0.006) and the occurrence of a severe infection during the first month of care (RR [95%CI] = 2.74 [1.27–5.92] *p* = 0.01) were the best predictors of one-year mortality (Table 3). The estimated survival median [IQR] time was 112 months [102–138]. Data on five-year patient survival (as a function of the two independent predictors of one-year mortality) are presented in Fig. 1.

**Table 2:** Univariate analysis for one-year mortality

Variable	RR [95%CI]	*p* value
Age	1.34 [1.01–1.68]^a^	0.03
Female	1.10 [0.94–1.28]	0.20
Diabetes mellitus	1.90 [0.90–4.02]	0.10
CCI	1.32 [1.02–1.71]	0.04
PS 3–4 vs. 0–2	2.90 [1.55–5.45]	< 0.001
PR3 vs. MPO	0.75 [0.31–1.78]	0.50
Peak SCr	1.13 [1.02–1.24]^b^	0.01
Dialysis	2.40 [1.26–4.55]	0.007
Serum albumin	0.94 [0.89–0.99]	0.02
Haemoglobin	0.67 [0.52–0.86]	0.002
Leukocyte	0.98 [0.89–1.07]	0.62
CRP	0.99 [0.95–1.04]	0.79
BVAS	1.04 [1.00–1.09]	0.05
FFS	3.48 [2.10–5.79]	< 0.001
CYC	0.50 [0.22–1.12]	0.09
MP pulses	0.83 [0.32–2.16]	0.70
PLEX	1.66 [0.75–3.66]	0.21
Co-trimoxazole	0.40 [0.20–0.84]	0.02
Infection at M1	3.47 [1.84–6.54]	< 0.001
Leukopenia at M1	2.05 [0.76–5.56]	0.15

*RR* relative risk, *CI* confidence interval, *CCI* Charlson Comorbidity Index, *PS* Performance Status, *PR3* proteinase 3, *MPO* myeloperoxidase, *SCr* serum creatinine, *CRP* C-reactive protein, *FFS* Five-Factor Score, *CYC* cyclophosphamide, *MP* methylprednisolone, *PLEX* plasma exchange

^a^per 5 years

^b^per 100 μmol/l

**Table 3:** Multivariable analysis for one-year mortality

Third variable added to the model	AIC	AUC	RR [95%CI]	*p* value
Infection at M1	122.1	0.799	FFS 2.57 [1.30; 5.09]	0.006
			Infection 2.74 [1.27; 5.92]	0.01
			Age 1.05 [0.99; 1.12]	0.09
PS 3–4 vs. 0–2	129.0	0.781	FFS 3.09 [1.51; 6.27]	0.002
			PS 1.92 [0.84; 4.35]	0.12
			Age 1.05 [0.99; 1.12]	0.13
Haemoglobin	126.7	0.761	FFS 3.06 [1.46; 6.40]	0.003
			Hg 0.82 [0.63; 1.06]	0.13
			Age 1.05 [0.99; 1.12]	0.09
Serum creatinine	130.1	0.759	FFS 2.98 [1.45; 6.13]	0.003
			SCr 1.09 [0.97; 1.22]	0.15
			Age 1.05 [0.99; 1.12]	0.07
Dialysis	130.2	0.758	FFS 2.96 [1.44; 6.08]	0.003
			Dialysis 1.66 [0.76; 3.60]	0.20
			Age 1.05 [0.99; 1.12]	0.10
Co-trimoxazole	127.5	0.752	FFS 2.98 [1.40; 6.34]	0.004
			CTZ 0.53 [0.23; 1.20]	0.13
			Age 1.04 [0.98; 1.11]	0.20
CCI	132.4	0.749	FFS 3.43 [1.71; 6.89]	0.0005
			CCI 1.12 [0.80; 1.57]	0.51
			Age 1.04 [0.97; 1.11]	0.22

Given the limited number of events for the primary endpoint (29 deaths) and Peduzzi et al.’s recommendations [26], we built several multivariable models with three variables (including age and the Five-Factor Score (FFS)) known to be prognostic factors. Next, each of the other candidate variables with a univariate *p* < 0.05 was added as the third variable. Even though age is included in the FFS score, we selected it because all patients were aged 65 or over. The best multivariable model was considered to be that with the lowest Akaike information criterion (AIC) and highest area under the curve (AUC)

*RR* relative risk, *CI* confidence interval, *PS* performance status, *Hg* haemoglobin, *CTZ* Co-trimoxazole, *CCI* Charlson comorbidity index

### Renal survival and relapses

**Fig. 2:**
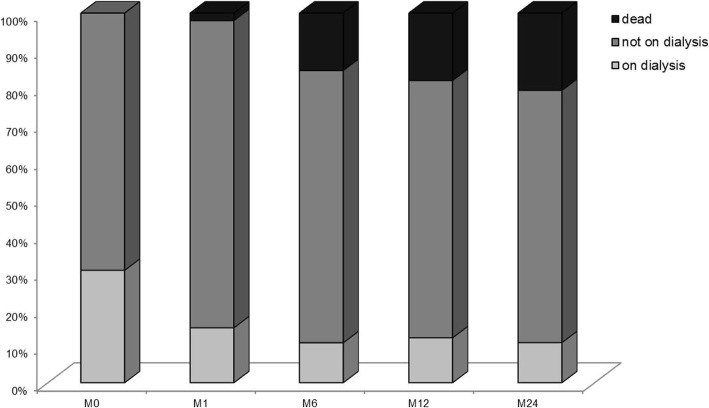
Outcomes during the first two years. The M24 analysis is based on data from 137 patients with at least 2 years of follow-up at the end of the study

After treatment initiation, the number of dialysed subjects fell from 45 at inclusion M0 to 22 after one month of care (21 patients had been weaned off dialysis, and 3 had died). Only two patients were weaned off dialysis after one month of care. At one year, 102 patients had conserved a degree of renal function, with a mean SCr level of 159 ± 67 μmol/l and a mean eGFR of 39.8 ± 16.7 ml/min/1.73 m^2^ (Fig. 2). The estimated proportion [95%CI] of patients on dialysis at 1, 3 and 5 years was 19% [12.6–25.0], 22% [15.2–28.8] and 27% [19.3–34.5], respectively. At baseline, the peak SCr concentration (HR [95%CI] = 1.27 [1.16–1.38]; *p* < 0.001), dialysis (HR [95%CI] = 2.66 [1.34–5.28]; *p* = 0.005) and the CCI (HR [95%CI] = 1.37 [1.11–1.68]; *p* = 0.002) were all independently associated with the risk of ESRD (Additional file 2: Table S2).

During the follow-up, a total of 43 (29%) patients relapsed. Thirty-two were major relapses, of which 29 involved the kidney (67%) and 4 involved the lungs. Eleven were minor relapses (ENT, joints, and eyes). Twenty-seven (63%) of these relapses occurred after the withdrawal of immunosuppression. The estimated median [95%CI] relapse-free survival time was 39.7 months [32.9–72.3]. In a multivariable analysis, PR3-ANCA was an independent risk factor for relapse (HR [95%CI] = 2.10 [1.15–3.82]; *p* = 0.01). Conversely, a high CCI (HR [95%CI] = 0.75 [0.59–0.96]; *p* = 0.02) and dialysis dependency (HR [95%CI] = 0.11 [0.01–1.17]; *p* = 0.07) were associated with a lower risk of relapse (Additional file 3: Table S3).

## Discussion

Our present results confirmed the severity of ANCA-associated renal vasculitis in older patients; the observed one-year mortality rate of 19.5% was much higher than the value of 2.6% recorded in the age-matched general population [11]. In agreement with the literature data, we found that most of the deaths occurred within the first six months and were primarily related to infectious diseases [6, 7]. Hence, when seeking to identify early predictors of death, we focused on baseline demographic characteristics and the first month of care. As reported previously, we found that disease severity markers (such as hypoalbuminemia, anaemia, a high FFS and altered renal function) are linked to “hard” outcomes [6, 9]. We (as others) found that the severity of renal disease at presentation correlated with the risk of ESRD [3, 12, 13]. Overall, 19% of the patients still had ESRD or had progressed to ESRD at one year. Lastly, 43 patients relapsed after a median of 40 months. Most of these relapses involved the kidney, and occurred after immunosuppression had been withdrawn. Hence, the presence of severe renal disease in the present study (as in the literature) was associated with a reduced risk of relapse [14, 15].

Our present results highlighted the impact of early infections on mortality. About 40% of the patients experienced a severe infection during the first 6 months of care - almost twice as many as the 24% reported by Little et al. at 12 months in a large cohort of AAV patients [7]. About a quarter of the patients developed a severe infection within the first month, and this event was independently associated with one-year mortality. Mc Gregor et al. have already shown that (i) the risk of death increases with the number of infections, and (ii) patients who experience a severe infection have a four-fold greater risk of death within 12 months [16]. Although we failed to show a statistically significant link between the therapeutic strategy and early mortality, it is clear that our study population (given the older age and the altered renal function) presented a higher risk of adverse drug reactions. Furthermore, the infection rate was highest during the first month and fell after the discontinuation of CYC and the steroid dose reduction.

The beneficial effect of immunosuppression on survival and ESRD was proved decades ago, and the response rate to immunosuppression is the same in elderly patients as it is in younger patients [17, 18]. However, the expected benefit of aggressive treatment is counterbalanced by the individual risk of adverse events and disease manifestations. For example, dialysis dependency after 1 month was associated with a low probability of renal recovery or disease relapse. Hence, in the absence of other organ-threating manifestations, the risk-benefit ratio should prompt a drastic reduction in the intensity of immunosuppressive therapy. In this respect, the KDIGO guidelines suggest discontinuing CYC after 3 months of dialysis dependency [19]. Given the early occurrence of infectious complications and deaths, it might be judicious to consider the early reduction or withdrawal of immunosuppressive therapy in some cases.

Reducing immunosuppression-related toxicity has been a constant concern over the last few decades. The association between corticosteroid exposure and infection is well established [20, 21], and the putative benefit of a reduced-dose glucocorticoid regimen in vasculitis is being tested in many ongoing randomized trials. A possible way forward has been addressed by the CORTAGE randomized trial of 140 over-65 patients with a mean baseline SCr of 233 μmol/l; treatment with low-dose corticosteroids and a fixed 500 mg IV pulse of CYC was associated with (i) a significant, early reduction in the adverse event rate and (ii) the same efficacy as a standard protocol [22]. These results confirm the importance of immunosuppressant-sparing strategies for improving the management of vasculitis in the elderly.

In parallel, strategies for preventing infection by the most common pathogens (comprehensive lung examinations, dipstick screening for urinary tract infection, the removal of non-essential intravascular catheters, etc.) might also help to reduce the incidence of severe infections. Given the study’s retrospective design and the long study period, it was difficult to clearly identify the specific reasons for the limited use of CTZ. Despite similar renal presentations, the attitude to prophylaxis varied from one centre to another. The use of CTZ was associated with a reduction in the early infection and one-year mortality rates, independently of the baseline serum creatinine level. Given the low rate of pneumocystis pneumonia (a single case), this result can be partly explained by CTZ’s ability to prevent bacterial infections [23] - suggesting a benefit of bacterial infection prophylaxis during the induction phase.

Our results also suggest that other patient-specific factors (such as the baseline ECOG Performance Status and the CCI) contribute to reduced tolerance of immunosuppression. Some previous studies have also found that comorbidity is correlated with lower patient and renal survival rates [24, 25].

Our study had several limitations. Firstly, the retrospective design may have introduced information and recall bias. However, the most important data were recorded during first month of care – a period during which the data collection was more reliable and clinical practice was relatively similar in the six centres. We found a one-year mortality rate of 19.5%, which is lower than the values of around 30% reported by previous studies of similar populations [3, 18]; this finding suggests that our study might not have been large enough to detect all the risk factors. Nevertheless, our demographic data and baseline presentations are similar to those observed in other series, and few patients were lost to follow-up. Cases of ANCA-negative pauci-immune glomerulonephritis were not included in the present study, which limits the extension of the present results to this population.

In conclusion, we found that over-65 patients presenting with AAV and renal involvement have a high mortality rate during the first months of care. Nevertheless, patients who survive the first year conserve a relatively good overall prognosis. When considering a variety of disease severity markers, an early-onset, severe infection (which affected about a quarter of the patients) was a strong predictor of one-year mortality. This finding should be taken into account during the initial steps in patient management. A reduction in immunosuppression, the early detection of infections, and systematic CTZ prophylaxis might help to reduce mortality in this population.

## Additional files

Additional file 1: **Table S1**. Causes of death within the first year of follow-up. (DOCX 16 kb)

Additional file 2: **Table S2**. Risk factors for end-stage renal disease. (DOCX 16 kb)

Additional file 3: **Table S3.** Risk factors for relapse. (DOCX 16 kb)

## Abbreviations

AAV: ANCA-associated vasculitis
AZA: Azathioprine
BVAS: Birmingham Vasculitis Activity Score
CCI: Charlson Comorbidity Index
CRP: C-reactive protein
CTZ: Co-trimoxazole
CYC: Cyclophosphamide
ECOG: Eastern Cooperative Oncology Group
eGFR: Estimated glomerular filtration rate
ESRD: End stage renal disease
FFS: 2009 Five-Factor Score
GPA: Granulomatosis with polyangiitis
MMF: Mycophenolate mofetil
MPA: Microscopic polyangiitis
MPO: Myeloperoxidase
PR3: Proteinase 3
RTX: Rituximab
SCr: Serum creatinine
