# Supplementary material for: Early predictors of one-year mortality in patients over 65 presenting with ANCA-associated renal vasculitis: a retrospective, multicentre study
Source: BMC Nephrol. 2018 Nov 9;19:317. doi: 10.1186/s12882-018-1102-3 (PMC6234782; doi:10.1186/s12882-018-1102-3)
Supplement: Supplementary file 1 — Table S1. Causes of death within the first year of follow-up. (DOCX 16 kb) [file 12882_2018_1102_MOESM1_ESM.docx]

| **Additional file 1: Table S1. Causes of death within the first year of follow-up.** | | |
| --- | --- | --- |
| **Time interval (months)** | **Cause** | **Precisions** |
| 0.9 | Infection | Septic shock (MRSA catheter infection) |
| 1 | Active vasculitis | Cardiac involvement |
| 1.1 | infection | Pneumonia |
| 1.1 | Active vasculitis | Diffuse alveolar haemorrhage |
| 1.3 | Active vasculitis | Gastrointestinal bleeding |
| 1.2 | Infection | Septic shock (MRSA pneumonia) |
| 1.3 | Cardiovascular | Stroke |
| 1.4 | Active vasculitis | Diffuse alveolar haemorrhage |
| 2 | Unknown | - |
| 2.2 | Infection | Spondylitis and peritonitis (*E. faecalis*, *E. cloacae*) |
| 2.6 | Infection | Pneumonia |
| 2.6 | Infection | Septic shock (invasive candidiasis) |
| 2.8 | Infection | Septic shock (peritonitis on ulcer perforation) |
| 3.1 | Infection | Septic shock (*Pseudomonas aeruginosa* pneumonia) |
| 3.2 | Infection | Septic shock |
| 3.7 | Cardiovascular | Cardiac arrest (ischemic heart disease) |
| 3.3 | Infection | Septic shock (pneumocystis) |
| 4.2 | Infection | Septic shock (*E. coli* pyelonephritis) |
| 4.2 | Infection | Septic shock (pneumonia, CD colitis) |
| 4.3 | Infection | Septic shock (MSSA pneumonia) |
| 4.3 | Infection | Septic shock (pneumonia) |
| 5.4 | Infection | Septic shock (peritonitis) |
| 5.7 | Infection | ESBL *E coli* pyelonephritis, CD colitis |
| 5.9 | Cardiovascular | Congestive heart failure |
| 6.7 | Infection | Pneumonia |
| 7.9 | Malignancy | Lymphoma with macrophage activation |
| 9.2 | Infection | Necrotizing fasciitis (*Pseudomonas aeruginosa*). CD colitis |
| 9.4 | Cardiovascular | Stroke |
| 11.8 | Infection | ARDS, Parainfluenza infection, and septic shock |
| MRSA, methicillin-resistant *Staphylococcus aureus*; MSSA, methicillin-susceptible *Staphylococcus aureus*; CD, *Clostridium difficile*; ARDS, acute respiratory distress syndrome; ESBL, extended spectrum beta-lactamase. *E. coli*, *Escherichia coli*; *E. cloacae*, *Enterobacter cloacae*; *E. faecalis*, *Enterococcus faecalis* | | |
