# Supplementary material for: Early predictors of one-year mortality in patients over 65 presenting with ANCA-associated renal vasculitis: a retrospective, multicentre study
Source: BMC Nephrol. 2018 Nov 9;19:317. doi: 10.1186/s12882-018-1102-3 (PMC6234782; doi:10.1186/s12882-018-1102-3)
Supplement: Supplementary file 2 — Table S2. Risk factors for end-stage renal disease. (DOCX 16 kb) [file 12882_2018_1102_MOESM2_ESM.docx]

| **Additional file 2: Table S2. Risk factors for end-stage renal disease** | | | | |
| --- | --- | --- | --- | --- |
|  | **Univariate analysis** | | **Multivariable analysis** | |
| **Variable** | **HR [95%CI]** | **p value** | **HR [95%CI]** | **p value** |
| Age | 1.05 [0.82–1.40]^φ^ | 0.63 |  |  |
| Female | 0.47 [0.25–0.88] | 0.02 |  |  |
| CCI | 1.41 [1.10–1.80] | 0.005 | 1.37 [1.11–1.68] | 0.002 |
| PR3 vs. MPO | 1.13 [0.60–2.15] | 0.71 |  |  |
| SCr | 1.32 [1.23–1.42]γ | <0.001 | 1.27 [1.16–1.38] | <0.001 |
| uPCR* | 1.35 [1.15–1.59] | <0.001 |  |  |
| Dialysis | 5.52 [3.07–9.90] | <0.001 | 2.66 [1.34–5.28] | 0.005 |
| Focal vs. other classes* | 0.00 [0.00–0.00] | <0.001 |  |  |
| Crescentic vs. mixed | 0.72 [0.21–2.48] | 0.61 |  |  |
| Crescentic vs. sclerotic | 0.95 [0.45–2.01] | 0.90 |  |  |
| BVAS | 1.04 [0.99–1.09] | 0.06 |  |  |
| CYC | 0.60 [0.31–1.15] | 0.12 |  |  |
| PLEX | 1.56 [0.83–2.93] | 0.16 |  |  |
| HR, hazard ratio; CI, confidence interval; CCI, Charlson Comorbidity Index; PR3, proteinase 3; MPO, myeloperoxidase; SCr, serum creatinine; uPCR, urinary protein-to-creatinine ratio; BVAS, Birmingham Vasculitis Activity Score; CYC, cyclophosphamide; PLEX, plasma exchange; γ per 100 µmol/l ; ^φ^per 5 years. Data included in the multivariable analysis: female, dialysis, SCr, CCI. *Not included in the multivariable model because of missing data. | | | | |
