# Supplementary material for: Early predictors of one-year mortality in patients over 65 presenting with ANCA-associated renal vasculitis: a retrospective, multicentre study
Source: BMC Nephrol. 2018 Nov 9;19:317. doi: 10.1186/s12882-018-1102-3 (PMC6234782; doi:10.1186/s12882-018-1102-3)
Supplement: Supplementary file 3 — Table S3. Risk factors for relapse. (DOCX 16 kb) [file 12882_2018_1102_MOESM3_ESM.docx]

| **Additional file 3: Table S3. Risk factors for relapse** | | | | |
| --- | --- | --- | --- | --- |
|  | **Univariate analysis** | | **Multivariable analysis** | |
| **Variable** | **HR [95%CI]** | **p value** | **HR [95%CI]** | **p value** |
| Age* | 0.94 [0.90–0.99]^φ^ | 0.01 |  |  |
| Female | 0.65 [0.36–1.17] | 0.15 |  |  |
| CCI | 0.70 [0.55–0.90] | 0.004 | 0.75 [0.59–0.96] | 0.02 |
| PR3 vs. MPO | 2.15 [1.22–3.79] | 0.008 | 2.10 [1.15–3.82] | 0.01 |
| SCr | 0.84 [0.72–0.97]γ | 0.017 |  |  |
| Dialysis baseline | 0.54 [0.26–1.12] | 0.10 |  |  |
| Dialysis at M1 | 0.10 [0.01–0.77] | 0.03 | 0.11 [0.01–1.17] | 0.07 |
| BVAS | 1.00 [0.95–1.05] | 0.93 |  |  |
| SCr at M12 | 0.76 [0.46–1.23] | 0.26 |  |  |
| IMS at M12 vs. no IMS | 1.86 [0.65–5.35] | 0.25 |  |  |
| IMS at M24 vs. no IMS | 1.76 [0.88–3.56] | 0.11 |  |  |
| HR, hazard ratio; CI, confidence interval; CCI, Charlson Comorbidity Index; PR3, proteinase 3; MPO, myeloperoxidase; SCr, serum creatinine; uPCR, urinary protein to creatinine ratio; BVAS, Birmingham Vasculitis Activity Score; CYC, cyclophosphamide; IMS, immunosuppression; γ per 100 µmol/l ; ^φ^per 5 years. Data included in the multivariable analysis: CCI, PR3, dialysis M1, SCr. *Not included in the multivariable model because CCI is adjusted for age. | | | | |
